# Puberty Onset and Positive Urgency Explain Diminished Returns of Family Income on Tobacco and Marijuana Use

**DOI:** 10.31586/ojp.2025.1141

**Published:** 2025-01-16

**Authors:** Shervin Assari, Babak Najand, Hossein Zare

**Affiliations:** 1 Charles R. Drew University of Medicine and Science, Los Angeles, CA, United States; 2 Marginalization-Related Diminished Returns (MDRs) Center, Los Angeles, CA, United States; 3 Johns Hopkins Bloomberg School of Public Health, Baltimore, MD, United States; 4 University of Maryland Global Campus (UMGC), Adelphi, MD, United States

**Keywords:** Puberty, Puberty Timing, Early Puberty Onset, Substance Use, Socioeconomic Status, Racial Disparities, Adolescence, Tobacco Use, Marijuana Use

## Abstract

**Background:**

Puberty is a crucial developmental milestone that involves significant physiological, emotional, and behavioral changes. Early puberty onset, influenced by both biological and social factors, is associated with an increased risk of engaging in substance use, such as tobacco and marijuana. While high family income is generally linked to delayed puberty onset and lower behavioral risks, these benefits may not be equally protective for Black youth due to the phenomenon of Minorities' Diminished Returns (MDRs). MDRs suggest that higher family income does not offer the same protective effects for Black youth as it does for White youth, potentially leading to earlier puberty and increased substance use among high-income Black adolescents.

**Objective:**

This study aimed to investigate whether early puberty onset and associated positive urgency (impulsivity) mediate the relationship between family income and the initiation of tobacco and marijuana use over a six-year follow-up period among adolescents. Additionally, the study examined whether the effects of family income on early puberty onset differ by race, testing the hypothesis that high-income Black youth would experience earlier puberty onset compared to their high-income White peers.

**Methods:**

Data were sourced from the Adolescent Brain Cognitive Development (ABCD) Study. Participants were 9-10-year-old adolescents at baseline, followed over a period of six years. Structural equation modeling (SEM) was used to assess whether early puberty onset mediated the effects of family income on substance use behaviors. Interaction terms between race and family income were included to test whether the impact of family income varies by race.

**Results:**

Early puberty onset and associated positive urgency partially explained the relationship between family income and the initiation of tobacco and marijuana use. High-income Black youth showed earlier puberty onset compared to their White counterparts. Earlier puberty onset then predicted higher positive urgency. These factors, in turn, were linked to higher rates of tobacco and marijuana initiation.

**Conclusions:**

This study provides additional evidence that the benefits of high family income do not extend equally to Black adolescents, particularly regarding delaying puberty onset and its consequences for substance use.

## 1. Introduction

Puberty is a critical developmental milestone that encompasses a series of physiological, emotional, and cognitive changes, often reshaping a young individual’s identity, social dynamics, and behavior [1]. These developmental shifts during adolescence are closely linked to health-related behaviors, including the initiation and escalation of substance use, such as tobacco, marijuana, and alcohol [2]. Earlier onset of puberty has been associated with an increased risk of engaging in risky behaviors, suggesting that the timing of puberty may have significant implications for adolescent health and well-being. The timing of puberty, however, is not solely determined by biological factors; it is heavily influenced by the social environment, socioeconomic status (SES) indicators such as family income, and exposure to various stressors, all of which intersect with race and gender.

Lower SES (e.g. family income) has long been associated with earlier onset of puberty, a phenomenon often linked to evolutionary adaptations. In environments characterized by adversity, earlier puberty may have provided an evolutionary advantage, allowing individuals to achieve reproductive maturity more quickly [3]. This "adaptive response" to stressful conditions, however, may come at a cost in contemporary societies, where earlier puberty is linked to adverse behavioral and health outcomes, including a heightened risk of substance use and emotional challenges such as depression. Additionally, research consistently shows that girls, compared to boys, tend to experience puberty earlier, highlighting the role of sex as a significant factor in developmental timing. The interplay between SES, stress, and sex has important implications for social epidemiology, as these factors shape trajectories of health and behavior, including vulnerability to substance use during adolescence.

Race, as both a social and structural determinant, further complicates the relationship between SES indicators such as family income and early puberty onset. Black and other minoritized populations are disproportionately exposed to stressors like economic instability, neighborhood disadvantage, and food insecurity—factors that are known to accelerate puberty. However, race not only influences exposure to these stressors but also affects the degree to which individuals benefit from protective resources like higher SES. This phenomenon, referred to as Minorities' Diminished Returns (MDRs) [4, 5], suggests that Black and other racialized groups often experience weaker positive effects of SES compared to their White counterparts. As a result, even in high-income families, Black children may not fully reap the developmental benefits of increased family income. For example, higher family income, often associated with delayed puberty and reduced behavioral risks for White youth, may not yield the same protective effects for Black youth due to ongoing exposure to racialized stress and discrimination.

One of the mechanisms by which low income and earlier puberty onset may elevate the risk for initiating tobacco and marijuana use is through their impact on impulsivity and inhibitory control. Low family income and earlier puberty are both linked to reductions in inhibitory control and increases in impulsivity, making adolescents more prone to risky behaviors. Positive urgency, a specific domain of impulsivity, is characterized by a tendency to act impulsively in response to intense positive emotions. This heightened impulsivity may lead to greater vulnerability to substance use, as adolescents with lower inhibitory control and higher positive urgency may struggle to resist engaging in high-risk behaviors like experimenting with tobacco and marijuana. The reduced capacity to regulate impulses in these contexts underscores the importance of addressing underlying social and developmental factors when aiming to mitigate substance use initiation in vulnerable groups.

This study aims to explore these complex dynamics using the Adolescent Brain Cognitive Development (ABCD) Study [6-14], a large and diverse sample that allows for a nuanced examination of the interactions between SES, race, and early puberty onset. Specifically, this study investigates whether early puberty onset mediates the relationship between family income and substance use behaviors, and whether the influence of family income on early puberty onset varies by race. We also hypothesized that early puberty onset effect on tobacco /marijuana initiation would be in part mediated via positive urgency. We hypothesize that, consistent with the MDRs framework, high-income Black youth will still experience earlier puberty compared to their high-income White peers, despite similar family income levels. This persistence of early puberty onset among high-income Black youth may lead to elevated risks of substance use, underscoring the need to consider both race and family income in understanding adolescent health disparities.

## 2. Methods

### 2.1. Study Design and Participants

This study utilized data from the Adolescent Brain Cognitive Development (ABCD) Study(6-14), the largest long-term study of brain development and child health in the United States. The ABCD Study follows over 11,000 children from diverse racial, ethnic, and economic backgrounds, starting at ages 9 to 10 years old, with annual follow-up assessments. The study is designed to provide a comprehensive understanding of cognitive, emotional, and social development during adolescence. Data were collected across 21 research sites in the United States using a multi-site, longitudinal cohort design. The dataset includes a wide range of measures, such as neuroimaging, cognitive assessments, substance use behaviors, and detailed demographic information.

### 2.2. Measures

#### Independent Variable: Family Income:

Family income was assessed based on parent-reported annual household income levels. Family income was categorized into several levels to examine its impact on various developmental and behavioral outcomes. Higher family income was hypothesized to be associated with a reduced likelihood of early puberty onset and lower substance use, with potential variations by race.

#### Mediator 1: Early Puberty Onset by Age 9/10:

Early puberty onset by age 9/10 was assessed using Tanner stage criteria, a validated measure of physical changes that mark the onset of puberty. This study focused on early puberty onset as a mediator in the relationship between family income and substance use. Higher Tanner scores indicated a more advanced stage of pubertal development by ages 9 to 10, identifying individuals with earlier onset of puberty.

#### Mediator 2: Positive Urgency:

Positive urgency, a trait linked to impulsive behavior under positive emotional states, was measured using the Urgency-Premeditation-Perseverance-Sensation Seeking-Positive Urgency (UPPS-P) Scale [32]. This scale was used to assess impulsivity driven by positive emotions, which may increase the risk of engaging in substance use. Positive urgency was examined as a mediator to explore its role in the pathway from early puberty onset to substance use.

#### Dependent Variables: Substance Use (Tobacco and Marijuana):

Substance use was captured through self-reported data on tobacco and marijuana use. Participants reported any initiation of tobacco or marijuana use over the past six years, allowing for the tracking of substance use behaviors from early to mid-adolescence. The validity of these self-reports was enhanced by additional questions regarding frequency and context of use.

#### Moderator: Race:

Race was self-identified and was limited to Black (coded as 1) or White (Coded as 0, and the reference category).

#### Covariates:

Demographic factors, including age, sex, ethnicity, and family structure (e.g., married or single-parent households), were included as covariates. These variables were controlled for in the analysis to account for their potential influence on early puberty onset and substance use.

### 2.3. Statistical Analysis

Structural equation modeling (SEM) was conducted to examine the proposed relationships between family income, early puberty onset by age 9/10, positive urgency, and substance use. SEM was selected for its ability to manage complex pathways, mediations, and interactions, providing a detailed understanding of direct, indirect, and moderation effects. In this analysis, early puberty onset and positive urgency were tested as mediators in the relationship between family income and the initiation of substance use (tobacco and marijuana). Interaction terms were included to explore whether the effects of family income on early puberty onset differed by race, focusing on the framework of Minorities' Diminished Returns (MDRs).

Model fit was assessed using established indices, including the Comparative Fit Index (CFI), the Tucker-Lewis Index (TLI), and the Root Mean Square Error of Approximation (RMSEA). Statistical significance was set at p < 0.05. All statistical analyses were performed using Stata 15.0 software.

### 2.4. Institutional Review Board (IRB) Approval

The ABCD Study received ethical approval from the Institutional Review Boards (IRBs) at University of California San Diego as well as other participating Universities, ensuring adherence to ethical standards in research with human subjects. All participants and their legal guardians provided informed consent or assent, following appropriate ethical protocols for minors. Participant privacy and confidentiality were strictly maintained throughout the study, in compliance with federal guidelines and institutional policies. This secondary analysis of ABCD data was exempt from review.

## 3. Results

Table 1 presents results from a structural equation model analyzing the Minorities' Diminished Returns (MDRs) of family income on the initiation of tobacco and marijuana use. The analysis includes pathways through which family income influences early puberty onset and positive urgency.

Higher family income is associated with a later onset of puberty (B = −0.049, SE = 0.018, 95% CI = −0.083, −0.014, p = 0.005). Latino ethnicity has a small but significant positive association with earlier puberty (B = 0.024, SE = 0.011, 95% CI = 0.001, 0.046, p = 0.039). Race (Black) does not show a significant direct effect on puberty onset (B = 0.021, SE = 0.033, 95% CI = −0.043, 0.085, p = 0.518). However, the interaction between race (Black) and family income significantly predicts earlier puberty (B = 0.062, SE = 0.029, 95% CI = 0.005, 0.118, p = 0.032). Being from a married family does not significantly influence pubertal onset (B = −0.020, SE = 0.013, 95% CI = −0.046, 0.006, p = 0.124). Age has a significant positive association with pubertal onset by age 9/10 (B = 0.113, SE = 0.011, 95% CI = 0.092, 0.134, p < 0.001). Male gender is negatively associated with earlier puberty onset (B = −0.082, SE = 0.011, 95% CI = −0.103, −0.061, p < 0.001).

Puberty onset by age 9/10 is significantly associated with an increase in positive urgency (B = 0.056, SE = 0.012, 95% CI = 0.032, 0.080, p < 0.001). Family income has a negative effect on positive urgency (B = −0.044, SE = 0.015, 95% CI = −0.074, −0.014, p = 0.005), while Latino ethnicity is positively associated with positive urgency (B = 0.026, SE = 0.011, 95% CI = 0.004, 0.048, p = 0.019). Black race is associated with higher positive urgency (B = 0.158, SE = 0.031, 95% CI = 0.098, 0.219, p < 0.001), and the interaction between race (Black) and family income shows a negative effect on positive urgency (B = −0.072, SE = 0.027, 95% CI = −0.126, −0.018, p = 0.009). Age is a strong predictor of positive urgency (B = 0.134, SE = 0.003, 95% CI = 0.127, 0.141, p < 0.001), and males have higher positive urgency (B = 0.091, SE = 0.011, 95% CI = 0.070, 0.112, p < 0.001).

Early puberty onset predicts increased likelihood of substance use (B = 0.039, SE = 0.013, 95% CI = 0.013, 0.064, p = 0.003). Positive urgency also contributes to higher substance use risk (B = 0.041, SE = 0.015, 95% CI = 0.012, 0.069, p = 0.006). Higher family income is associated with reduced substance use (B = −0.162, SE = 0.018, 95% CI = −0.197, −0.128, p < 0.001). The interaction between race (Black) and family income is positively associated with substance use (B = 0.175, SE = 0.032, 95% CI = 0.113, 0.238, p < 0.001). Latino ethnicity does not significantly affect substance use (B = −0.018, SE = 0.013, 95% CI = −0.043, 0.007, p = 0.156). In contrast, being Black is associated with significantly lower substance use (B = −0.231, SE = 0.036, 95% CI = −0.301, −0.160, p < 0.001). Age positively predicts substance use (B = 0.039, SE = 0.004, 95% CI = 0.030, 0.047, p < 0.001), while being male has a marginally non-significant protective effect (B = −0.020, SE = 0.012, 95% CI = −0.044, 0.004, p = 0.098).

## 4. Discussion

The primary aim of this study was to examine the relationship between family income, early puberty onset, and substance use among adolescents, with a particular focus on potential racial differences. Using the Adolescent Brain Cognitive Development (ABCD) Study's diverse sample, we tested whether early puberty onset mediates the effects of family income on substance use behaviors, including tobacco and marijuana. Additionally, we sought to determine whether race moderates the influence of family income on early puberty onset, exploring the hypothesis that high-income Black youth would exhibit earlier puberty compared to their White peers, aligning with the framework of Minorities' Diminished Returns (MDRs) [15-20].

Our findings supported all initial hypotheses. Early puberty onset was a significant mediator in the relationship between SES and substance use behaviors, specifically tobacco and marijuana. While higher SES indicators such as family income were generally associated with later puberty and lower substance use in the full sample, these effects were not uniform across racial groups. Black adolescents from high-income families demonstrated earlier puberty than their White counterparts, which was linked to increased rates of tobacco and marijuana use over a six-year follow-up period. These results align with the MDRs framework, suggesting weaker protective effects of high SES for Black youth.

Socioeconomic status (SES) indicators such as family income plays a central role in determining health and behavioral outcomes during adolescence. Typically, higher SES indicators such as family income are associated with delayed puberty and reduced likelihood of engaging in substance use, largely due to greater access to resources, less exposure to stress, and a safer social environment. In our study, higher family income was generally protective against early puberty onset and subsequent substance use, which is consistent with prior research. However, the differential impact of family income by race highlights the limitations of assuming uniform benefits across diverse populations.

A significant interaction emerged between family income and race, where Black adolescents from higher SES backgrounds experienced earlier puberty compared to their White counterparts of similar family income. This pattern suggests that the advantages typically conferred by higher family income may not extend fully to Black youth. These findings reflect broader patterns of racial disparities in health outcomes, where Black individuals often face additional stressors and barriers, even at similar levels of economic resources. Factors such as racial discrimination, exposure to chronic stress, and neighborhood characteristics may play critical roles in explaining why family income effects differ by race.

The observed racial differences in the impact of family income align with the concept of Minorities' Diminished Returns (MDRs) [21-25], which posits that the protective benefits of many SES indicators are attenuated for minoritized groups. Several root causes likely contribute to these diminished returns, including systemic racism, differential access to high-quality education and healthcare, exposure to greater psychosocial stress, and the challenges of navigating predominantly White or affluent spaces [4,5]. These structural and contextual factors diminish the potential health benefits associated with higher family income among Black adolescents, making it harder for economic resources alone to fully protect against early puberty onset and its consequences [31].

Early puberty has been linked to various mental and behavioral risks for adolescents, as the rapid biological and hormonal changes may outpace their emotional and cognitive development [33-35]. Adolescents who experience early puberty are often at an increased risk of developing mental health issues such as depression, anxiety, and low self-esteem [36-38]. The early onset of puberty can also expose adolescents to heightened social pressures, including bullying, peer rejection, or premature engagement in adult-like behaviors [39,40]. Behaviorally, early-maturing adolescents are more likely to engage in risky behaviors, such as substance use, early sexual activity, and delinquent behavior, potentially as a way to navigate or cope with these social pressures [41-43]. Additionally, early puberty is associated with spending more time in higher-risk contexts and forming connections with riskier peer groups, further amplifying their vulnerability to adverse outcomes [44-48]. These risks are often compounded by environmental factors, such as familial stress, peer influences, or cultural expectations, underscoring the importance of providing supportive and protective environments for early-maturing youth [49.^50^].

### 4.1. Implications

These findings have significant implications for public health interventions and policies. Efforts to reduce substance use among adolescents may need to account for the differential impacts of SES by race, particularly in addressing the unique vulnerabilities of Black youth from high-income families. Interventions should consider not only socioeconomic factors but also the broader social and environmental contexts, including racism and discrimination, that shape adolescent development. Strategies that address structural inequalities, improve neighborhood conditions, and promote racial equity are crucial to mitigating the risk of early puberty onset and substance use among Black youth.

### 4.2. Limitations

This study has several limitations. First, while the ABCD dataset provides a diverse and large sample, self-reported data on substance use and family income may be subject to biases, including social desirability and recall biases. We only investigated family income and did not investigate other SES indicators such as wealth. Additionally, the cross-sectional nature of some measurements limits the ability to draw causal conclusions. Although we examined early puberty onset as a mediator, other unmeasured factors such as family dynamics, neighborhood characteristics, or genetic influences may also play a role. Lastly, the generalizability of these findings may be limited to populations similar to those in the ABCD study.

### 4.3. Future Research Directions

Future research should further explore the mechanisms underlying the MDRs [26-30] of a wide range of SES indicators on early puberty onset and substance use, with a focus on identifying modifiable factors that could reduce these disparities. Longitudinal studies examining the impact of structural and contextual factors—such as exposure to discrimination, neighborhood characteristics, and school environments—would provide deeper insights into the pathways linking SES, early puberty onset, and substance use among different racial groups. Additionally, qualitative studies may shed light on the lived experiences of high-income Black adolescents, offering a nuanced understanding of how racialized stress affects developmental outcomes.

## 5. Conclusions

In conclusion, this study provides compelling evidence that the benefits of high family income do not extend equally to Black adolescents, particularly regarding delaying puberty onset and its consequences for substance use. The MDRs framework highlights the need to move beyond simplistic understandings of family SES as a uniform protective factor, emphasizing the importance of context, race, and systemic inequalities. Addressing these disparities requires comprehensive and culturally sensitive approaches that consider the unique challenges faced by minoritized populations, aiming to reduce racial inequities in adolescent development and health outcomes.

## Figures and Tables

**Figure 1.**
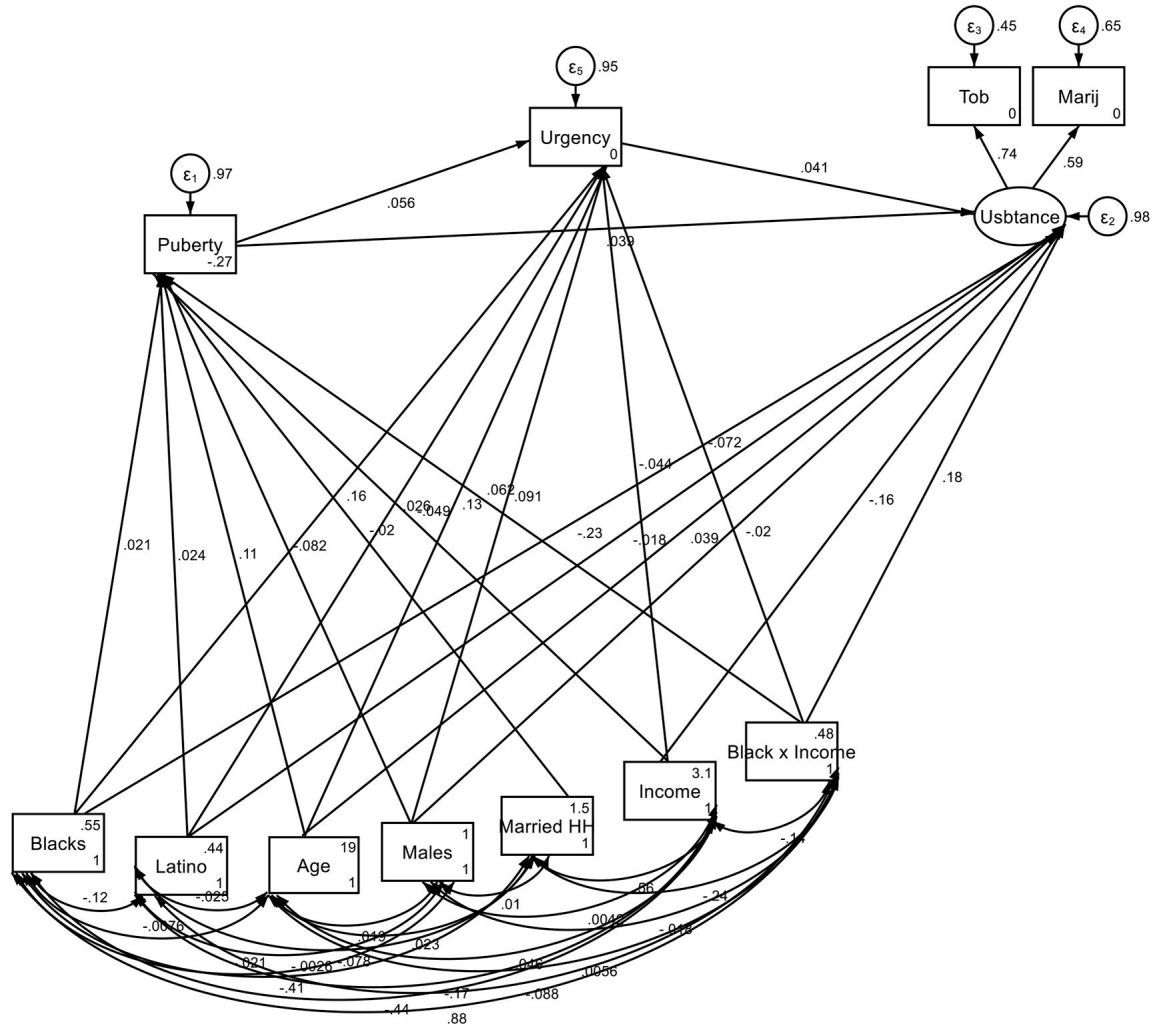
Summary of Structural equation modeling (SEM) on Minorities' Diminished Returns (MDRs) of family income on tobacco/marijuana use initiation via Minorities' Diminished Returns (MDRs) of family income on early puberty onset and positive urgency **Note:** Tob: tobacco; Marij: Marijuana, Married HH: Married Household; Urgency: Positive Urgency

**Table 1.** Summary of Structural equation modeling (SEM) on Minorities' Diminished Returns (MDRs) of family income on tobacco/marijuana use initiation via Minorities' Diminished Returns (MDRs) of family income on early puberty onset and positive urgency

			B	SE	95% CI		p
							
Age	→	Pubertal Onset by Age 9/10	0.113	0.011	0.092	0.134	< 0.001
Male	→	Pubertal Onset by Age 9/10	−0.082	0.011	−0.103	−0.061	< 0.001
Family Income	→	Pubertal Onset by Age 9/10	−0.049	0.018	−0.083	−0.014	0.005
Ethnicity (Latino)	→	Pubertal Onset by Age 9/10	0.024	0.011	0.001	0.046	0.039
Race (Black)	→	Pubertal Onset by Age 9/10	0.021	0.033	−0.043	0.085	0.518
Race (Black) x Family Income	→	Pubertal Onset by Age 9/10	0.062	0.029	0.005	0.118	0.032
Married Family	→	Pubertal Onset by Age 9/10	−0.020	0.013	−0.046	0.006	0.124
							
Pubertal Onset by Age 9/10	→	Positive Urgency	0.056	0.012	0.032	0.080	< 0.001
Age	→	Positive Urgency	0.134	0.003	0.127	0.141	< 0.001
Male	→	Positive Urgency	0.091	0.011	0.070	0.112	< 0.001
Family Income	→	Positive Urgency	−0.044	0.015	−0.074	−0.014	0.005
Ethnicity (Latino)	→	Positive Urgency	0.026	0.011	0.004	0.048	0.019
Race (Black)	→	Positive Urgency	0.158	0.031	0.098	0.219	< 0.001
Race (Black) x Family Income	→	Positive Urgency	−0.072	0.027	−0.126	−0.018	0.009
							
Pubertal Onset by Age 9/10	→	Tobacco/ Marijuana	0.039	0.013	0.013	0.064	0.003
Positive Urgency	→	Tobacco/ Marijuana	0.041	0.015	0.012	0.069	0.006
Age	→	Tobacco/ Marijuana	0.039	0.004	0.030	0.047	< 0.001
Male	→	Tobacco/ Marijuana	−0.020	0.012	−0.044	0.004	0.098
Family Income	→	Tobacco/ Marijuana	−0.162	0.018	−0.197	−0.128	0.000
Ethnicity (Latino)	→	Tobacco/ Marijuana	−0.018	0.013	−0.043	0.007	0.156
Race (Black)	→	Tobacco/ Marijuana	−0.231	0.036	−0.301	−0.160	< 0.001
Race (Black) x Family Income	→	Tobacco/ Marijuana	0.175	0.032	0.113	0.238	< 0.001
